# Application progress of single-cell sequencing technology in mesenchymal stem cells research

**DOI:** 10.3389/fcell.2023.1336482

**Published:** 2024-01-09

**Authors:** Hao Li, Yusong Wang, Gehua Zhu, Qimin Ma, Shengyu Huang, Guanghua Guo, Feng Zhu

**Affiliations:** ^1^ Medical Center of Burn Plastic and Wound Repair, The First Affiliated Hospital, Jiangxi Medical College, Nanchang University, Nanchang, China; ^2^ Department of Burns, The First Affiliated Hospital, Naval Medical University, Shanghai, China; ^3^ Department of Critical Care Medicine, Shanghai East Hospital, Tongji University School of Medicine, Shanghai, China

**Keywords:** single-cell sequencing, mesenchymal stem cells, surface markers, differentiation trajectories, immunomodulation

## Abstract

Single-Cell Sequencing (SCS) technology plays an important role in the field of Mesenchymal Stem Cells (MSCs) research. This paper comprehensively describes the application of SCS technology in the field of MSCs research, including (1) SCS enables more precise MSCs characterization and biomarker definition. (2) SCS reveals the prevalent gene expression heterogeneity among different subclusters within MSCs, which contributes to a more comprehensive understanding of MSCs function and diversity in developmental, regenerative, and pathological contexts. (3) SCS provides insights into the dynamic transcriptional changes experienced by MSCs during differentiation and the complex web of important signaling pathways and regulatory factors controlling key processes within MSCs, including proliferation, differentiation and regulation, and interactions mechanisms. (4) The analytical methods underpinning SCS data are rapidly evolving and converging with the field of histological research to systematically deconstruct the functions and mechanisms of MSCs. This review provides new perspectives for unraveling the biological properties, heterogeneity, differentiation potential, biological functions, and clinical potential of MSCs at the single-cell level.

## 1 Introduction

Mesenchymal Stem Cells (MSCs) represent a subtype of adult stem cells found in various tissues within the human body, distinguished by their capacity for self-renewal and multi-lineage differentiation potential. Among the diverse array of stem cell types, MSCs stand as the most extensively researched and widely applied. Originating from the mesodermal layer during early embryonic development, MSCs fall under the category of multipotent stem cells (Keating, 2006). In 1968, Russian scientist Alexander Friedenstein made the pioneering discovery of MSCs within bone marrow and successfully cultivated them *in vitro* (Friedenstein et al., 1968; Friedenstein et al., 1974). It was not until 1999 that Pittenger et al. achieved a landmark milestone by inducing MSCs to differentiate into adipocytes, osteocytes, and chondrocytes *in vitro*, thereby affirming the multi-lineage differentiation potential of MSCs (Pittenger et al., 1999). In 2006, the International Society for Cellular Therapy (ISCT) standardized the definition criteria for MSCs, establishing the following key characteristics (Dominici et al., 2006): (1) adherence to plastic culture dishes under standard culture conditions, (2) expression of specific surface markers, with CD105, CD73, and CD90 expression exceeding 95%, and the absence of CD45, CD34, CD14 or CD11b, CD79α or CD19, and HLA-DR surface molecules, (3) demonstrated ability to differentiate into adipocytes, osteocytes, and chondrocytes. Given the myriad potential applications, MSCs have become a focal point of both research endeavors and clinical practices. However, it is imperative to acknowledge that despite their considerable potential, the clinical application of MSCs remains subject to certain challenges and controversies (Andrzejewska et al., 2019; Lukomska et al., 2019). Variations exist among MSCs sourced from different tissues, individuals, and environmental conditions, manifesting as heterogeneity in terms of cell morphology, surface markers, proliferation and differentiation potential, as well as immunomodulatory capabilities. These issues engender challenges related to cell source selection, control of expansion and differentiation, long-term efficacy, and safety in the context of MSCs research and application (Ullah et al., 2015; Musial-Wysocka et al., 2019; Rodriguez-Fuentes et al., 2021). Further research and exploration are necessitated to address these complexities comprehensively.

**FIGURE 1:**
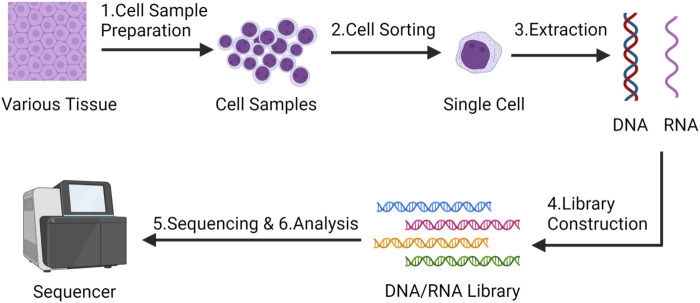
The main steps of single-cell sequencing technology - Created by BioRender.com.

Traditional population sequencing technology is difficult to capture the cellular heterogeneity and diversity among MSCs, in stark contrast to conventional bulk sequencing techniques, SCS distinguishes itself by its capacity to investigate the genetic information of individual cells (Figure 1), thereby unearthing intercellular disparities and diversities. SCS can unveil the gene expression characteristics and cellular state alterations of individual MSCs. This portends that the SCS technology analysis process will yield more intricate and precise data, thereby facilitating a profound comprehension of mesenchymal stem cells. Based on this, SCS technology has extended many applications in MSCs research (Ullah et al., 2015; Zheng et al., 2020; Qu et al., 2021; Zhang et al., 2022; Lin et al., 2023): 1. Defining the characteristics and markers of MSCs: using SCS can help to define the characteristics and markers of MSCs, specifically identify and localize MSCs, complete classification and clustering, and deepen the understanding of their lineage hierarchy. 2. Study MSCs heterogeneity: SCS can reveal the gene expression heterogeneity of different cells in the MSCs population. It can determine the differences between different cell subsets, as well as the gene expression changes within the cells. Studying the heterogeneity of MSCs can help us to better understand their differences and improve their therapeutic efficacy, which is of great help in reducing the uncertainty of MSCs as therapeutic agents, while identifying and isolating MSCs subsets with specific functions and characteristics, which can help to understand the function and diversity of MSCs in development, regeneration and disease. So as to promote the development of individualized medicine. 3. Characterize the differentiation potential and trajectory of MSCs: With SCS, the transcriptome changes of MSCs during differentiation can be studied. This helps to determine the multilineage differentiation potential of MSCs and reveals the transcriptome dynamics of MSCs differentiating into different cell types. 4. Detection of MSCs signaling pathways and regulators: SCS can identify important signaling pathways and regulators in MSCs. It can reveal the regulatory network and key transcription factors of MSCs and help to understand the mechanisms of proliferation, differentiation and regulation of MSCs. 5. Study the immunomodulatory ability of MSCs: SCS can also study the immunomodulatory ability of MSCs. By analyzing the interaction between MSCs and immune cells and the expression of regulatory molecules, the function and mechanism of MSCs in the immune system can be revealed.

In general, the main steps of SCS include (Andrews et al., 2021; Zhang et al., 2021):

1. Cell sample preparation and isolation: obtaining a cell sample from tissue, blood or other sources. Usually, cells are prepared by a series of pretreatment steps, such as centrifugation, filtration, digestion, etc., to obtain a suspension of individual cells. Various technical approaches have been employed for isolating single cells, encompassing techniques such as serial dilution, micromanipulation, fluorescence-activated cell sorting (FACS), immunomagnetic separation (IMS), laser capture microdissection (LCM), and microfluidic platforms (Chi, 2014). Each method presents distinct merits and drawbacks. Therefore, it is advisable for researchers to carefully select the most suitable technique for isolating single cells based on their unique experimental conditions.
2. Single cell sorting: Single cell sorting technology is used to separate cells one by one and place them in individual cell Wells or reaction droplets to ensure that each cell is sequenced independently;
3. Cell lysis and DNA/RNA extraction: The DNA or RNA of individual cells is extracted for subsequent sequencing analysis.
4. DNA/RNA library construction: library construction of extracted DNA or RNA, which includes processing, labeling and amplification of DNA/RNA fragments;
5. High-throughput sequencing: high-throughput sequencing of the library to obtain cellular DNA/RNA sequence information;
6. Data analysis: The obtained SCS data were analyzed, the typical workflow for single-cell RNA sequencing analysis begins with the generation of a count or Unique Molecular Identifier (UMI) matrix. Given the inherent noise in single-cell data, the initial processing involves quality control, normalization, feature selection, and dimension reduction of the matrix. Subsequent downstream analyses encompass tasks such as clustering, trajectory inference, cell-type annotation, and dataset integration. Through SCS, researchers can better understand the function and diversity of cells and reveal important mechanisms during cell development and disease occurrence.

This article aims to review the application of SCS technology in the study of MSCs, and provide new insights into the biological characteristics, heterogeneity, differentiation ability, biological function, and clinical application of MSCs at the single-cell level.

## 2 Application of SCS in the isolation, identification, heterogeneity, and subclusters classification of MSCs

MSCs have been identified and successfully isolated from a variety of tissues, such as adipose tissue, lung, liver, bone marrow, umbilical cord, synovium, amniotic fluid, fetal blood, dental pulp, skeletal muscle and even from the circulatory system (Ullah et al., 2015; Han et al., 2019). As previously elucidated, ISCT proposes minimum criteria for defining MSCs, providing a standardized framework for defining and characterizing MSCs through their functional and phenotypic properties. Although this definition is precise enough, other published studies suggest that the biological identification of MSCs remains divisive (Dominici et al., 2006; Keating, 2006). ISCT also suggested that novel surface markers that may be discovered in the future may lead to modifications of these criteria. differences in isolation and purification of MSC may result showing varied immunomodulatory properties. Numerous subsequent research results have demonstrated that various research laboratories have introduced new terminology and classification criteria. Nonetheless, the molecular expression profiles and biological functions of these cells exhibit similarity, and SCS technology can unveil characteristic molecular expression patterns of MSCs. This capability assists in more precise definition and identification of MSCs while enabling their differentiation from other cell types. Therefore, SCS plays a pivotal role in delineating the distinguishing characteristics, identifying markers, and contributing to the exploration of heterogeneity within the field of MSCs.

### 2.1 Define the characteristics and markers of MSCs

Various studies have reported specific or partially overlapping combinations of surface markers for MSCs. In 2011, Mafi P et al. provided a comprehensive overview of research findings on the cell surface characteristics of adult mesenchymal stem cells (Mafi et al., 2011). Various studies have presented contradictory information regarding specific cell surface markers such as CD10, CD34, CD44, CD45, CD49d, and CD106. The disparities in MSC surface marker expression can be attributed to factors such as the tissue origin of MSCs and variations in cell proliferation stages and culture processes. Current evidence for several cell surface markers is conflicting and insufficiently informative, requiring further research in this area. Therefore, one of the challenges in distinguishing or precisely defining MSCs lies in the varying combinations of cell surface markers. Through SCS, researchers can accurately screen the surface markers and characteristic genes of MSCs, further refining their definition. In conjunction with existing standard definitions, the newly discovered surface markers and marker genes can provide more precise and specific methods for the identification and isolation of MSCs.

In this section, we will emphasize SCS-related research concerning the frequently utilized tissue sources for MSCs, more surface markers of MSCs are shown in Table 1. Wang et al. employed the most commonly used tissue sources for obtaining MSCs in clinical settings. This included samples from bone mar-row (*n* = 3), adipose tissue (*n* = 3), umbilical cord (*n* = 2), and dermis (*n* = 3) (Wang et al., 2021a). They con-ducted single-cell RNA sequencing (scRNA-seq) to generate a comprehensive atlas comprising over 130,000 individual transcriptomes of MSCs. They characterized dis-tinct tissue-specific by identifying characteristic genes, numerous genes categorized as transcription factors, ligands, ligand-binding receptors, and cytokines exhibit high ex-pression levels in tissue-specific subclusters. Nonetheless, the specific functions of co-expressed transcription factors have yet to be elucidated. Furthermore, fibroblasts were considered as aged MSCs, akin to MSCs, all fibroblasts demonstrated positivity for CD73, CD90, and CD105, and negativity for CD14, CD34, CD45, CD19, and HLA-DR (Soundararajan and Kannan, 2018). The lack of distinctive markers presenting a challenge in accurately studying their respective functions. This research provided single-cell dataset encompassing MSCs from diverse tissues, CD106, CD146, ITGA11, SSEA-4, and GD-2 have been shown to be MSCs specific, provides a foundation for future endeavors molecular markers and phenotypes capable of distinguishing fibroblasts from MSCs.

**TABLE 1:** Surface markers of human MSCs from different tissue sources.

Source	Cell surface markers		References
	Positive	Negative	
Bone marrow	CD29, CD44, CD73, CD90, CD105, Sca-1, STRO-1	CD11b, CD14, CD19, CD34, CD45, CD86, Ia, HLA-DR	Stewart et al. (1999), El-Ansary et al. (2012), Otsuru et al. (2013), Zhou et al. (2019), Gao et al. (2021), Medrano-Trochez et al. (2021)
Adipose tissue	CD13, CD29, CD44, CD73, CD71, CD090, CD105, CD146, CD166, MHC-I, STRO-1	CD11b, CD13, CD14, CD19, CD31, CD34, CD45, HLA-DR	Zuk et al. (2002), Mushahary et al. (2018), Zhou et al. (2019), Bunnell (2021)
Umbilical cord	CD29, CD44, CD73, CD90, CD105, CD166, CD362, SH2, SH3, Tra-1–60, Tra-1–81, SSEA-1, SSEA-4	CD10, CD14, CD31, CD34, CD45, CD106, HLA-DR	Secco et al. (2008), Sibov et al. (2012), Mushahary et al. (2018), Gonzalez et al. (2020), Zhang et al. (2021b), Medrano-trochez et al. (2021)
Wharton’s jelly	CD13, CD29, CD90, CD105, CD106	CD3, CD4, CD14, CD15, CD34, CD45, HLA-DR	Hou et al. (2009), Watson et al. (2015), Chen et al. (2023)
Dental tissues	CD13, CD29, CD44, CD73, CD90, CD105, CD106, CD146, CD166, CD271, STRO-1, OCT-4, SSEA-4	CD3, CD8, CD11b, CD14, CD15, CD19, CD33, CD34, CD45, CD71, CD117, HLA-DR	Grawish (2018), Chen Et al. (2021), Cabana-Munoz Et al. (2023)
Placenta	CD73, CD90, CD105, CD146	CD34, CD45	Raynaud et al. (2012), Beeravolu et al. (2017)
Endometrium	CD44, CD73, CD90, CD105, HLA-ABC	CD14, CD34, CD45, CD133, HLA-DR	Cheng et al. (2017)
Labial, salivary and lacrimal gland	CD13, CD29, CD73, CD44, CD90, STRO-1	CD34, CD45, HLA-DR	Rotter et al. (2008), Li et al. (2021), Jaffet et al. (2023)
Peripheral blood	CD44, CD73, CD90, CD105, CD166, SSEA-4, Vimentin	CD14, CD19, CD34, CD45, HLA-DR	Fu et al. (2015), Wang et al. (2022b)
Synovium and Synovial fluid	CD10, CD13, CD44, CD49, CD73, CD90, CD105, CD147, CD166, STRO-1	CD14, CD20, CD31, CD34, CD45, CD62, CD68, CD106, CD113, CD117, HLA-DR, ALP	Morito et al. (2008), Isobe et al. (2016)

In another scRNA-seq analysis of human bone marrow MSCs (BMSCs) conducted by Xie et al. a cohort comprising a total of 15 healthy volunteers was recruited for MSC collection (Xie et al., 2022). Analysis of scRNA-seq and flow cytometry data revealed that bone marrow MSCs exhibited positivity for CD29, CD44, and CD105, while being negative for CD14, CD34, and CD45. The researchers identified three distinct MSC clusters based on their specific expression patterns, namely, the CD26^+^ stem cell subclusters, the CMKLR1+ functional subclusters, and the proliferation subclusters. Gao et al. concentrate on recent advancements in identifying and classifying BMSCs (Gao et al., 2021). They review SCS studies that report the classic and newly discovered roles of BMSCs and their connections in the context of both physiological and pathological states. Their objective is to identify a connection among the overlapping of BMSCs, skeletal stem cells, and adipocyte lineage cells, the results show that BMSCs express CD105, CD73, and CD90 but not CD45, CD34, CD14, or CD11b, CD79α, or CD19 or HLA-DR surface molecules.

As reported by Zhou, adipose-derived stem cells (ADSCs) and bone marrow-derived stem cells (BMSCs) were isolated and cultured from the same donor. Single-cell and bulk-cell assays were employed to identify and compare the characteristics of ADSCs and BMSCs at both single-cell and bulk-cell levels. ADSCs should be positive for the following markers: CD10, CD13, CD29, CD34, CD44, CD49d, CD54, CD59, CD73, CD90, CD105, and CD166, and the following markers should be negative: CD11b, CD14, CD19, CD31, CD45, CD56, CD106, CD146, HLA-DR. And according to the research report by Bunnell et al., the expression of CD34 decreases as ADSCs undergo continuous passages. Additionally, there is an absence of expression of CD80, CD86, CD40, and their ligand CD40L in these cells.

Comparative findings in related SCS studies of umbilical cord (UC) and Wharton’s Jelly (WJ) MSCs highlight distinct expression profiles and heterogeneity within these adjacent tissue sources (Zhang et al., 2021). They examined MSCs obtained directly from Wharton’s jelly and compared them to cultured UC-MSCs, the SCS data reveal unique transcriptomic signatures, specific markers, and diverse cellular subclusters within both UC and WJMSCs: the freshly isolated MSCs were negative for CD31, CD34, CD45, or CD11b, and markers reveal that freshly isolated MSCs can be categorized into two distinct cell populations based on the level of CD73 expression. The cell population expressing low levels of CD73 also exhibits CD200 expression but does not express CD106 or CD146; cultured cells were positive for CD73, CD90, CD105, and CD44, negative for CD34, CD45, CD11b, and HLA-DR. shedding light on their potential functional differences and therapeutic implications. Consequently, the surface marker expression pattern of uncultured MSCs differs from that of cultured UC-MSCs. This distinction underscores the importance of considering the dynamic nature of surface marker expression during the culture and expansion of MSCs. Further exploration of these comparative analyzes contributes to a comprehensive understanding of MSCs derived from different sources and aids in harnessing their therapeutic potential effectively (Barrett et al., 2019).

Drawing upon these revelations, the application of SCS technology augments our capacity to attain a comprehensive and precise grasp of the intricate attributes and discerning markers that define MSCs. This results in a universally applicable spectrum of positive and negative cell surface markers. This standardized compendium establishes a robust and standardized method for the characterization of MSCs populations that can be effectively applied in in vitro and *in vivo* settings. Such advancements bestow heightened accuracy upon the identification of MSCs, culminating in the refinement of isolation methods. This, in turn, fostering transformative strides in the application of MSCs for therapeutic endeavors.

### 2.2 Reveal the heterogeneity of MSCs

Recent research indicates that the diversity in the gene expression profiles of MSCs intended for therapeutic purposes stems from interindividual heterogeneity. This variability is influenced by factors such as age, sex, tissue specificity, as well as disparities arising from distinct isolation methods, passages, and subsets of MSCs. These sources of heterogeneity hold significant implications for the functionality and application of MSCs (Xu et al., 2017). In the quest to discern specific cell subsets within this heterogeneous MSCs population, researchers have consistently sought out distinctive cell surface markers and molecular features. In addition to identifying these surface markers, SCS can help to identify and study genes related to MSCs function. By revealing the transcriptome differences and expression heterogeneity of different cells in the MSCs population, it helps to identify and understand the differences between MSCs subsets. Analyzing the single-cell transcriptome data of MSCs in a large number of cells can find the gene expression patterns related to specific functions (such as self-renewal, multi-directional differen-tiation potential, immune regulation, etc.) of different subsets of MSCs (Zhang et al., 2022). Thus, we can better understand the biological characteristics of MSCs, perform clustering and classi-fication analysis of MSCs, and classify similar cells into the same subclusters. This helps to identify and define different cell types and subsets within MSCs, further re-vealing their heterogeneity. The classification of MSCs subclusters identified by SCS method is shown in Table 2.

**TABLE 2:** Classification and characteristics of MSCs subclusters reported in studies.

Source	Subclusters names	Surface markers or marker genes or function	References
Bone Marrow	C0: BMSC-specific	LEPR, CXCL12, CXCL16	Wang et al. (2021a)
	C2: Stemness subclusters	SOX4, GAS1, DPP4	Xie et al. (2022)
	C4: Functional subclusters	Cytokines and factors associated with osteogenic and adipogenic differentiation	Xie et al. (2022)
	C5 and C6: Proliferative subclusters	proliferative and cell-cycle-related genes	Xie et al. (2022)
Adipose	C2: ADSCs-specific	COL15A1, COL5A3	Wang et al. (2021a)
	C3: ADSCs-specific	CDCP1, IL33	Wang et al. (2021a)
Umbilical Cord	C4: UCMSC-specific	KRT8, KRT18, LMO3	Wang et al. (2021a)
Dermis	C5: DMSCs-specific	CCL13, NGFR	Wang et al. (2021a)
	C7: DMSCs-specific	TFP2A, TBX5	Wang et al. (2021a)
	C9: DMSCs-specific	IGF1, TMEM176A/B	Wang et al. (2021a)
Wharton’s Jelly	C1: Potential stem cells	TOP2A, MKI67, E2F1, CCNA2	Sun et al. (2020)
	C2: Lineage differentiation	ID4, SCX, COL11A1, PPARG, CEBPD	Sun et al. (2020)
	C3	Extracellular structural tissues, developmental processes, skin development, muscle contraction	Chen et al. (2023)
	C4: Immunomodulatory	CCL2, GCSF (CSF3), VEGF, IL-7	Chen et al. (2023)
	CD73 high expressing MSCs	Inflammation, muscle proliferation, cell differentiation, and oxidative stress	Zhang et al. (2021b)
	CD73 low expressing MSCs	(ECM) synthesis, bone and cartilage growth	Zhang et al. (2021b)

As described above, Wang et al., following stringent quality control and data standardization, a comprehensive map was constructed, encompassing over 130,000 single MSCs transcriptomes. These transcriptomes were derived from 11 normal donors aged between 22 and 46 years, representing various tissues including adipose, bone marrow, dermis, and umbilical cord. The data was stratified into 12 distinct clusters (C0-C11) based on the identification of highly variable genes. Differential gene expression analysis revealed unique characteristics for MSCs within each cluster: The BMSC-specific C0 subset primarily expressed well-known MSC marker genes such as LEPR, CXCL12, and CXCL16. The ADSCs-specific subclusters C2 expressed COL15A1, COL5A3, while the C3 subset expressed CDCP1 and IL33. UCMSC-specific subclusters C4 exhibited expression of KRT8, KRT18, and LMO3. DMSCs-specific subclusters C5 expressed CCL13, NGFR; C7 expressed TFP2A, TBX5; and C9 expressed IGF1, TMEM176A/B. These genes are linked to the process of lineage differentiation, tissue repair, and immunomodulation. These findings highlight the unique expression profiles and characteristics of MSCs within specific subclusters, shedding light on the tissue-specific features and potential functional roles of MSCs in different contexts. Xie et al. captured total of 6,998 cells and then divided cells into 10 clusters (C0-C9) using K-means methods, and used marker genes and further bioinformatics analysis to identify three major subsets of BMSCs, namely, stem-like, functional, and proliferative subsets (Xie et al., 2022). C2, characterized by elevated levels of stemness markers such as SOX4, GAS1, and DPP4, was identified as the stemness subclusters. On the other hand, C4, which showcased the expression of cytokines and factors associated with osteogenic and adipogenic differentiation, was labeled as the functional subclusters. Furthermore, C5 and C6, both demonstrating the expression of proliferative and cell-cycle-related genes, were subcategorized into proliferative subclusters 1 and 2, underscoring their proliferative nature and involvement in cell cycle processes. These categorizations enhance our understanding of the distinct functional states and characteristics within the mesenchymal stem cell population. At the same time, in a SCS analysis of WJMSCs, 12 469 cells were divided into 3 clusters, C1 (12.6%), C2 (61.1%) and C3 (26.3%), and the results showed only the C1 subclusters of WJ-MSCs may be real stem cells and the MSCs probably do not have immune privilege (Leng et al., 2022).

It was noted that MSCs exhibit varying expression levels of CD73. Specifically, the CD73 high expressing MSCs were found to express genes associated with diverse biological activities, including inflammation, muscle proliferation, cell differentiation, and oxidative stress response. On the other hand, CD73 low expressing MSCs were observed to express genes related to extracellular matrix (ECM) synthesis, bone and cartilage growth, as well as glucose metabolism (Zhang et al., 2021). These differential gene expression profiles shed light on the functional diversity and potential of MSC subclusters based on CD73 expression levels.

Through single-cell RNA sequencing of 61,296 mesenchymal stem cells (MSCs) derived from both bone marrow and Wharton’s jelly, six distinct subclusters (C0-C5) were identified (Zhang et al., 2022). Among these, two subclusters were recognized for their potential roles in self-renewal and immune regulation. The C1 subclusters exhibited robust active proliferation characteristics, featuring high expression levels of proliferation markers such as TOP2A, MKI67, E2F1, and the cell cycle regulator CCNA2. Furthermore, C1 cells demonstrated high expression of characteristic markers associated with perivascular mesodermal progenitor cells, including NG2/CSPG4, CD146/MCAM, and NES, suggesting their potential for stemness. On the other hand, the C2 subclusters displayed characteristic expression of genes related to three lineage differentiations: ID4, SCX, COL11A1, PPARG, and CEBPD. These findings deepen our understanding of the functional diversity and potential of MSC subclusters derived from bone marrow and Wharton’s jelly. Two additional research teams have also investigated the heterogeneity of WJMSCs using single-cell sequencing (SCS) analysis.

In one study, the samples were categorized into 6 clusters (C0-C5) (Sun et al., 2020). Notably, the upregulated genes in the C3 subclusters were significantly enriched in extracellular structural tissues, developmental processes, skin development, muscle contraction, and related pathways. In the C4 subclusters, elevated expression levels of cytokines like CCL2, GCSF (CSF3), VEGF, and IL-7 were observed, indicating its potential for immunomodulatory therapeutic applications. And researchers further isolated and sorted CD142+ WJMSCs. Comparative analysis with fibroblast scratch experiments demonstrated that CD142+ WJMSCs exhibited superior *in vitro* proliferation ability and enhanced “wound healing” potential. These findings underscore the diverse functional attributes and potential applications of different subclusters within WJMSCs. In a separate study, researchers delved into WJMSCs, partitioning them into 13 clusters (C0-C12) (Chen et al., 2023). Each cluster displayed unique gene expressions linked to specific biological functions: Proliferation-Related subclusters (C0, C2, C3, C7) highly expressed proliferation-related genes: UBE2C, TOP2A, HMGB2, CDC20, PCNA, HIF1A. Niche‐supporting subclusters (C1, C4, C11) marked by genes: ACTA2, TGM2, FOS, TGFB1, FLNA, COL3A1. Metabolism-Related Cluster (C10) characterized by genes: S100A10, PDLIM1, CAV1. Biofunctional‐type subclusters (C5, C8, C12) expression of genes: UBE2S, TAGLN, TPM1, TMSB4X. Regenerative and Immunomodulatory subclusters (C6, C9) labeled by genes: B4GALT1, HEG1, CXCL3, CXCL1, HMOX1. The study also encompassed a temporal analysis, providing insights into subpopulation distribution in the P3 generation and shedding light on the trajectory and occurrence of these subpopulations.

Increasing evidence highlights the presence of multiple subclusters within MSCs, each characterized by specific surface markers. SCS has emerged as a powerful tool to elucidate gene expression variations within the MSC population. Comparative analysis of transcriptome data from different cells enables the identification of characteristic gene expression differences among diverse cell subclusters, ultimately unraveling the heterogeneity inherent to MSCs. Further research endeavors are essential to precisely define these subclusters using specific biomarkers and delineate their biological functions. This understanding will enable the selection of distinct application scenarios and methodologies based on the functional characteristics of these subclusters, thereby optimizing the utilization of MSCs by leveraging their diverse functions and unlocking their maximum potential.

## 3 Application of SCS in the direction of MSCs biological function

### 3.1 Applications in the field of MSC differentiation research

MSCs exhibit remarkable versatility, capable of differentiating into osteogenic, chondrogenic, adipogenic, and myogenic lineages. The effective utilization of differentiated MSCs in tissue regeneration depends on their capacity to maintain the intended cell identity throughout the entire process. MSCs exhibit remarkable versatility, capable of differentiating into osteogenic, chondrogenic, adipogenic, and myogenic lineages. The successful application of differentiated MSCs in tissue regeneration hinges on their ability to sustain the desired cell fate throughout the process. the impact of cytokines, chemokines, and transcription factors, as well as microenvironmental changes, on MSC differentiation, play crucial role in guiding MSCs toward specific mesenchymal lineages, a deeper understanding of the intricate regulation of MSC differentiation through various levels of transcription factors during the differentiation process is essential. The potential of MSCs to differentiate into specific mesenchymal lineages hinges on the precise upregulation or repression of lineage-specific genes. These changes occur during differentiation, influenced by specific signaling pathways and interactions with other transcription factors acting as co-regulators. Single-cell sequencing technology enables the analysis of the transcriptome and regulatory factors involved in MSC differentiation, further enhancing our ability to predict and identify the differentiation trajectories of MSCs. Continued research in this area is essential for unlocking the full therapeutic potential of MSCs. Single-cell sequencing technology can track and analyze the transcriptome changes of MSCs during differentiation, thereby revealing their multi-directional differentiation potential and differentiation trajectory (Lin et al., 2023). This helps to understand the mechanisms of MSCs differentiation and the transcriptional regulatory networks that develop into specific cell types. To identify and analyze key transcription factors and regulatory networks in MSCs. These transcription factors and regulatory networks play important roles in maintaining the stemness properties of MSCs and regulating cell fate decisions (Xu et al., 2017).

A single-cell RNA-seq analysis comparing data from umbilical cord, bone marrow, synovial tissue, and adipose tissue-derived MSCs (Hou et al., 2021). They successfully identified three major cell subgroups across all MSC samples, namely, osteo-MSCs, chondro-MSCs, and adipo/myo-MSCs. The study revealed that certain stemness-related genes, such as AMIGO2, CLDN1, LRRC17, and SLC22A3, were significantly upregulated in UC-MSCs (umbilical cord-derived MSCs) compared to MSCs from other tissue sources. Additionally, immune-regulatory genes like AREG, CSF3, CCL20, and IL6 were also significantly elevated in UC-MSCs, suggesting that umbilical cord MSCs may be the optimal source for immune modulation and tissue repair. Furthermore, the research analyzed the differentiation induction response of these three major subgroups and found that when the direction of differentiation matched the induction direction, the subgroups expanded and differentiated, while in the opposite scenario, subgroup numbers decreased. The study also compared the transcription factor (TF) regulatory activity of these three subgroups before and after induction in different lineage specifications. For instance, in chondrogenic differentiation, FOSL2, ATF5, FOXF1, and HES7 played critical roles, with FOSL2 potentially being of paramount importance. In osteogenic induction, MAFF, FOXO1, and MXI1 were pivotal, and FOXO3 upregulated several genes, including LEPR, CTGF, ITGA5, and COL5A2. In adipogenic induction, CEBPA, PPARG, and STAT5A were also crucial, and these transcription factor networks jointly regulated several genes associated with adipogenesis, such as ADIPOQ, PLIN4, FABP4, and FABP5. According to a study by Lin and colleagues, which combined single-cell RNA sequencing and Cell Tagging (Lin et al., 2023), it was found that as MSCs from adipose tissue underwent *in vitro* passages, their heterogeneity increased. Through the tracking provided by Cell Tagging, the study demonstrated that early human adipose-derived stem cells ADSCs spontaneously differentiated into two distinct cell subgroups. One subgroup exhibited robust proliferation and differentiation potential, while the other was inherently associated with osteogenic differentiation right from the beginning. This osteogenic-specific cluster seems to emerge during the early passage stages. The study also unveiled potential candidate genes for osteogenic differentiation, with SERPINE2 being highlighted. The activation of SERPINE2 was shown to significantly enhance BMP-mediated bone formation, emphasizing its substantial impact on osteogenic differentiation. Wang and colleagues conducted an analysis of the differentiation potential of Bone Marrow-Derived Mesenchymal Stem Cells (BMMSCs) through single-cell transcriptome profiling. They annotated these cells into distinct subgroups based on known cellular markers or functional genes, categorizing them as pre-osteoblastic subcluster (Cluster C1, expressing osteogenic markers, including collagen type I and ALPL 36,37); pre-adipocytic subcluster (Cluster C2, expressing adiponectin and MGP); pre-chondrocytic subcluster (Cluster C6, expressing CD56 and WIF1); and terminally differentiated cells that do not express differentiation markers (Clusters C3-C5). Further analysis of differentially expressed genes (DEGs) specific to each subgroup revealed high expression of CD146 in the pre-osteoblastic subcluster, APOD (Apolipoprotein D) in the pre-adipocytic subcluster, and OMD (Osteomodulin) in the pre-chondrocytic cells. Moreover, it was determined that DEGs within the pre-chondrocytic subcluster were enriched in signaling pathways including PI3K-Akt, MAPK, Ras, Rap1, TGF-beta, Apelin, and Hippo. CD56^+^ pre-chondrocytic cells demonstrated the ability to undergo chondrogenesis and myogenesis, with these processes potentially being regulated by the TGF-β, Apelin, and Ras/Rap1 signaling pathways (Wang et al., 2021b).

Additionally, Li’s team, through the use of single-cell sequencing technology, has reported a novel type of MSC (mesenchymal stem cell) derived from human adipose tissue (Li et al., 2018). They have named this new type of MSC “Mature Adult-Derived Stem Cells” (M-ADSCs). These cells consistently exhibit high expression of markers such as Flk1, CD29, CD44, CD105, and CD166 (≥95%), while showing low expression of CD106, CD31, CD34, CD45, and HLA-DR (≤5%). These cells exhibit distinctions from previously documented MSCs in that they exist in a state characterized by open chromatin, demonstrating epigenetic pluripotency and expressing pluripotency markers such as MYC, KLF4, and GMNN. The majority of genes associated with germ layer specification undergo modification marked by H3K4me3 or co-modification involving both H3K4me3 and H3K27me3. Through single-cell level analysis of their surface markers and differentiation capabilities, it has been demonstrated that M-ADSCs can be induced to become functional stem cells and can transition to a pluripotent state at the pre-induction stage without the need for a reprogramming process.

Moreover, the amalgamation of SCS technology with pseudotime analysis not only enables a more precise delineation of the differentiation pathways undertaken by MSCs but also provides deeper insights into their developmental trajectories. In summary, leveraging single-cell sequencing technology for the analysis of the differentiation potential of MSCs serves as a valuable tool for researchers to gain a more comprehensive understanding of the intricate mechanisms underlying MSC behavior. This approach not only enhances our knowledge of MSC differentiation but also offers insights into their broader role in various biological processes and potential therapeutic applications.

### 3.2 Interaction between MSCs and immune system

MSCs possess the remarkable capability to modulate the activity of various immune cells, such as T cells, B cells, natural killer (NK) cells, dendritic cells, and macrophages. They achieve this regulation by releasing a diverse array of cytokines and chemokines that exert a profound influence on the immune response. The interaction between MSCs and the immune system constitutes a highly intricate and dynamic process, which has been a focal point of extensive research efforts (Le Blanc and Mougiakakos, 2012; Shi et al., 2018). SCS technology plays a crucial role in unveiling the genes responsible for immunomodulation that are expressed by MSCs during these interactions. By comparing the transcriptome data of MSCs with that of immune cells, researchers can pinpoint the key genes and signaling pathways involved in MSC-mediated immune regulation. This detailed analysis contributes to a more profound understanding of the molecular mechanisms through which MSCs affect immune cell activation, inflammatory responses, and immune homeostasis. This advanced method allows for the comprehensive examination of the interaction between MSCs and immune cells and the expression patterns of immunomodulatory molecules. It proves instrumental in shedding light on the mechanisms behind MSC-mediated immune regulation (Leng et al., 2022).

Based on different disease models, the immunomodulatory effects and therapeutic effects of MSCs from different sources have been shown to be different. According to existing studies, MSCs exhibit pro-inflammatory and anti-inflammatory properties, however, these properties are exerted by different cell subsets (Uccelli et al., 2008; Wang et al., 2022). Li’s research team has made significant contributions in the field of uncovering the immunomodulatory mechanisms of MSCs in the treatment of ALI (acute lung injury) through single-cell RNA sequencing. In 2020, the research team reported the mechanism of MSC treatment for ALI. MSCs exert their therapeutic effect by reducing the infiltration of CD8^+^ T cells, especially Ly6C+ CD8^+^ T cells, into the lungs and decreasing their expression of pro-inflammatory factors (Zhu et al., 2020). ScRNA-seq revealed that Ly6C+ CD8^+^ T cells exhibited a more activated phenotype, and their expression of pro-inflammatory factors decreased following treatment with MSCs. These pro-inflammatory factors are particularly abundant in immune chemotaxis. In the same year, the research team also reported that they used scRNA-Seq to identify four distinct lung B cell clusters in ALI mice (Feng et al., 2020). Furthermore, they demonstrated that the classification of B cell clusters was independent of whether MSC treatment was administered. The research findings indicate that in the early stages of ALI, MSCs exhibit inhibitory effects on chemokines such as Ccl3, Ccl4, and Cxcl10 in lung B cells, as well as on signaling pathways including TNF, Th17, and NF-κB. During the recovery phase of ALI, MSCs also suppress the expression of immunoglobulins in lung B cells. These discoveries may contribute to elucidating the mechanisms of MSC treatment for ALI. It is suggested that the impact of MSCs on antibody production by lung B cells might require a longer observation period. In 2021 (Liu et al., 2021), the team delineated the dynamic functions and phenotypic profiles of mononuclear phagocytes recruited in ALI mice and those subjected to MSC treatment. They precisely identified eight subsets of mononuclear phagocytes recruited to LPS-challenged lungs. In ALI mice not subjected to MSC treatment, the lungs exhibited recruitment of both Ly6ChiCD38+ and Ly6ClowCD38+ monocytes on day 3 post LPS administration, coinciding with an upregulation in the secretion of neutrophil chemokines. Ly6ChiCD38+ monocytes underwent differentiation into M1 macrophages by day 3, subsequently progressing into CD38^+^ monocyte-derived dendritic cells (mo-DCs) by day 7. Ly6ClowCD38+ monocytes differentiated into CD11b+CD38^+^ dendritic cells (DCs) by day 7. Following MSC treatment in ALI mice, there was a significant reduction in mortality rates. MSCs mitigated the quantity of M1 macrophages on day 3 and decreased the secretion of neutrophil chemokines. Furthermore, MSCs diminished the population of CD38^+^ mo-DCs and CD11b+CD38^+^ DCs on day 7, thereby suppressing the antigen presentation process. Notably, the recruited mononuclear phagocyte subsets displaying heightened CD38 levels exhibited an activated phenotype, leading to the secretion of elevated levels of cytokines and chemokines. In 2023, they investigated neutrophil characteristics in ALI mouse lung tissue after MSC treatment using single-cell RNA sequencing. The study revealed six clusters of neutrophils, including activated, aged, and circulatory subtypes (Feng et al., 2023). Activated neutrophils exhibited higher chemotaxis, reactive oxygen species (ROS) production, and NADPH oxidase scores compared to aged neutrophils. MSC treatment shifted activated neutrophils toward an aged neutrophil phenotype by upregulating CD24 expression, resulting in reduced inflammation through decreased chemotaxis, ROS production, and NADPH oxidase activity. These findings collectively contribute to a better understanding of the mechanisms underlying MSC treatment in ALI and its impact on immune cells, particularly CD8^+^ T cells, B cells, and neutrophils.

In a mouse model of bleomycin-induced lung fibrosis, Tang and colleagues studied the molecular and cellular behavior of human umbilical cord-derived mesenchymal stem cells (hucMSCs). They tracked the behavior of MSCs in the lungs by transfecting them with GFP (Green fluorescent protein) (Tang et al., 2021). Using single-cell RNA sequencing, they examined how hucMSCs influenced the gene expression profile of macrophages following bleomycin treatment. Their research identified a subset of macrophages known as interferon-sensitive macrophages (IFNSM), which increased the secretion of CXCL9 and CXCL10, thereby recruiting more Treg cells to the injured lungs. This provides new insights into how hucMSCs interact with macrophages and exert their immunomodulatory functions during pulmonary fibrosis repair. In a study investigating the therapeutic effects of ADMSCs on pulmonary fibrosis., using a mouse model of pulmonary fibrosis induced by bleomycin and GFP-labeled mouse ADSCs, they explored the interactions between ADSCs and pulmonary cells at the single-cell level. They successfully recovered ADSCs injected into the trachea and performed scRNA-seq alongside pulmonary cells. ADSC treatment significantly altered the transcriptional profiles and composition of pulmonary cells, particularly macrophages. They delved into the signaling pathway interactions between ADSCs and pulmonary cells, revealing potential regulatory pathways, including NGR, ANNEXIN, HGF, and PERIOSTIN. The data suggest that injected ADSCs increased the population of Trem2+ anti-inflammatory pulmonary macrophages, leading to a reduction in pulmonary inflammation and fibrosis (Rahman et al., 2022).

Zhu et al. conducted a phase II clinical trial involving 29 patients per group, using a randomized, single-blind, placebo-controlled design to validate prior findings on MSCs infusion in COVID-19 patients. Single-cell RNA sequencing analysis of peripheral blood revealed a newly identified subpopulation of VNN2+ hematopoietic stem/progenitor cell-like (HSPC-like) cells expressing CSF3R and PTPRE, which mobilized post-MSC infusion. Additionally, various immune cells showed upregulation of chemokine genes (CX3CR1 and L-selectin). MSC treatment influenced B cell subsets, and both *in vivo* and *in vitro*, it enhanced T cell expression of the costimulatory marker CD28. The study underscores MSC’s role in maintaining immune homeostasis and improving the prognosis of COVID-19 patients (Zhu et al., 2021).

## 4 SCS combined with omics methods in MSCs research

The rapid advancement of multi-omics SCS technology enables the simultaneous, comprehensive analysis of the genome, transcriptome, epigenome, and proteome at the single-cell level. This allows for a more systematic and thorough examination of individual cells (Dai and Shen, 2022). Proteomics can study the expression, modification and interaction of proteins in cells. By combining single-cell sequencing and proteomics, we can compare the protein levels of different genes in MSCs and understand the relationship between gene expression and protein expression. This helps to reveal the process of post-transcriptional regulation in cells, the regulatory mechanism of protein function, and the interaction network between proteins. Metabolomics studies the composition and changes of intracellular metabolites, which can provide information on the metabolic state and metabolic pathways of cells. Combining single-cell sequencing with metabolomics can reveal the metabolic characteristics of MSCs and the regulation of metabolic pathways under different gene expression patterns. This helps to understand the energy metabolism, material transformation and metabolic adaptability of cells, as well as the interrelationship between gene expression and metabolism.

Single-cell RNA sequencing pseudo-time analysis is an analytical method used to understand the cell states and developmental trajectories within single-cell data. This analysis helps determine the position of individual cells in their developmental or functional context, beyond mere time points in the data. Through pseudo-time analysis, it becomes possible to identify genes and signaling pathways expressed at specific developmental time points or states, aiding in the comprehension of functional changes during cellular development. By analyzing characteristic expression patterns in single-cell data, such as changes in gene expression levels, it is feasible to infer the pseudo-temporal order of cells along their developmental trajectory. To gain deeper insights into the distinct subtypes of BM-MSCs and their differentiation relationships, Wang et al. employed a strategy to reconstruct the developmental trajectory. This involved inferring dynamic gene expression patterns at various developmental stages. By ordering cell type clusters along a pseudotime continuum, the researchers unveiled a spectrum of gene expression transitioning from early to late stages of BM-MSC differentiation (Wang et al., 2021b).

The combination of single-cell sequencing and temporal analysis can help us understand the dynamic changes during cell differentiation and development. Chen et al. conducted a single-cell sequencing and temporal analysis (ST analysis) on four sections obtained from different parts of the same umbilical cord (UC). They compared the gene expression profiles of these sections with corresponding single-cell RNA sequencing (scRNA-seq) samples, establishing correlations between spatial gene expression and tissue image information. The analysis identified spatial clusters based on cluster-specific marker genes, revealing molecular heterogeneity between maternal and fetal segments. DEG sequencing highlighted distinct transcription characteristics, with maternal segments expressing higher levels of ECM-related genes such as COL1A1, COL1A2, COL18A1, BGN, and ELN. Functional enrichment analysis of these DEGs showed significant enrichment in ECM organization, cell-junctional organization, focal adhesion, and ECM-receptor interactions. In contrast, fetal segments exhibited enrichment in gene sets related to skin development, epidermal development, and neutrophil activation, suggesting their potential role as a crucial mesenchymal stem cell (MSC) source for healing skin wounds (Chen et al., 2023).

## 5 Summary

SCS can reveal the genetic expression characteristics and cellular state changes of individual MSCs. It can be used in MSC research to map the lineage hierarchy, gene expression heterogeneity, identify and isolate MSCs with specific functions and characteristics, describe the differentiation potential and differentiation trajectory of MSCS. With the rapid development of SCS data analysis methods and omics research, multiple omics information is integrated, and data quality control, preprocessing, abnormal molecular identification, interaction prediction, enrichment analysis, path analysis, network construction, and more advanced or centralized analysis are used to systematically understand the function and mechanism of MSCs. With the rapid development of MSC clinical application, single-cell sequencing is used to detect the transcriptome differences between MSCs from different individuals, including gene expression levels, regulatory factor activity and signaling pathway activation, which is helpful to understand the characteristics and potential differences, potential and treatment response of MSCs from different individuals. It provides a basis for individualized regenerative medicine and tissue engineering, so as to realize the development of individualized medicine. In terms of predicting treatment response, the transcriptome data of MSCs are analyzed, the influence of different treatment regimens on MSCs and the potential therapeutic response are analyzed, and the best strategy for individualized treatment is determined to improve the therapeutic effect and predict the treatment response of patients. To reveal the changes in gene expression related to specific diseases in MSCs, identify disease-related genes and pathways, and provide new targets and strategies for individualized diagnosis and treatment of diseases, so as to develop individualized treatment strategies. To select the appropriate source of MSCs for patients, optimize the culture conditions of MSCs, and apply them in individualized treatment.

## Abbreviations

SCS, Single-Cell Sequencing; MSCs, Mesenchymal Stem Cells; ISCT, International Society for Cellular Therapy; ALI, Acute lung injury; GFP, Green fluorescent protein; LPS, Lipopolysaccharides; ST analysis, Single-cell sequencing and temporal analysis; UC, Umbilical cord; ECM, Extra Cellular Matrix.
